# Ischemia-guided vs routine non-culprit vessel angioplasty for patients with ST segment elevation myocardial infarction and multi-vessel disease: the IAEA SPECT STEMI trial

**DOI:** 10.1007/s12350-022-03108-z

**Published:** 2022-10-25

**Authors:** Ganesan Karthikeyan, Amalia Peix, Niveditha Devasenapathy, Amelia Jimenez-Heffernan, Saif-ul Haque, Carlo Rodella, Raffaele Giubbini, Erick Alexanderson Rosas, Elgin Ozkan, Yung Jih Felix Keng, João Vitola, Dragana Sobic-Saranovic, Manoj Soni, Leonardo López, Lázaro O. Cabrera, Santiago Camacho-Freire, Ana Manovel-Sanchez, Hesham Naeem, Shazia Fatima, Roberto Rinaldi, Isabel Carvajal-Juarez, Kerim Esenboga, Maurizio Dondi, Diana Paez

**Affiliations:** 1 Department of Cardiology, All India Institute of Medical Sciences, New Delhi, India; 2 Department of Nuclear Medicine, Cardiology and Cardiovascular Surgery Institute, Havana, Cuba; 3 The George Institute for Global Health, New Delhi, India; 4 Hospital Universitario Juan Ramón Jiménez, Huelva, Spain; 5 Nuclear Medicine, Oncology and Radiotherapy Institute (NORI), Islamabad, Pakistan; 6 Health Physics Department, ASST-Spedali Civili, Brescia, Italy; 7 Nuclear Medicine, University of Brescia and Spedali Civili Brescia, Brescia, Italy; 8 National Institute of Cardiology Ignacio Chavez, Mexico City, Mexico; 9 Department of Physiology, Faculty of Medicine, Universidad Nacional Autonoma de Mexico, Mexico City, Mexico; 10 Department of Nuclear Medicine, Medical School, Ankara University, Ankara, Turkey; 11 Department of Cardiology, National Heart Centre Singapore, Singapore, Singapore; 12 Quanta Diagnóstico por Imagem, Curitiba, Brazil; 13 Faculty of Medicine, University of Belgrade, Belgrade, Serbia; 14 Center for Nuclear Medicine, University Clinical Centre of Serbia (UCCS), Belgrade, Serbia; 15 Department of Interventional Cardiology, Cardiology and Cardiovascular Surgery Institute, Havana, Cuba; 16 Rawalpindi Institute of Cardiology, Rawalpindi, Pakistan; 17 Hospital de Cardiología Centro Médico Nacional Siglo XXI, Mexico City, Mexico; 18 Department of Cardiology, Medical School Ankara University, Ankara, Turkey; 19 Nuclear Medicine and Diagnostic Imaging Section, Division of Human Health, Department of Nuclear Sciences and Applications, International Atomic Energy Agency, Vienna, Austria; 20 Cardiothoracic Sciences Centre, All India Institute of Medical Sciences, 24, 7th Floor, New Delhi, 110029 India

**Keywords:** Myocardial ischemia and infarction, SPECT, MPI

## Abstract

**Background:**

In patients with multi-vessel disease presenting with ST elevation myocardial infarction (STEMI), the efficacy and safety of ischemia-guided, vs routine non-culprit vessel angioplasty has not been adequately studied.

**Methods:**

We conducted an international, randomized, non-inferiority trial comparing ischemia-guided non-culprit vessel angioplasty to routine non-culprit vessel angioplasty, following primary PCI for STEMI. The primary outcome was the between-group difference in percent ischemic myocardium at follow-up stress MPI. All MPI images were processed and analyzed at a central core lab, blinded to treatment allocation.

**Results:**

In all, 109 patients were enrolled from nine countries. In the ischemia-guided arm, 25/48 (47%) patients underwent non-culprit vessel PCI following stress MPI. In the routine non-culprit PCI arm, 43/56 (77%) patients underwent angioplasty (86% within 6 weeks of randomization). The median percentage of ischemic myocardium on follow-up imaging (mean 16.5 months) was low, and identical (2.9%) in both arms (difference 0.13%, 95%CI − 1.3%–1.6%, *P* < .0001; non-inferiority margin 5%).

**Conclusion:**

A strategy of ischemia-guided non-culprit PCI resulted in low ischemia burden, and was non-inferior to a strategy of routine non-culprit vessel PCI in reducing ischemia burden. Selective non-culprit PCI following STEMI offers the potential for cost-savings, and may be particularly relevant to low-resource settings.

(CTRI/2018/08/015384).

**Supplementary Information:**

The online version contains supplementary material available at 10.1007/s12350-022-03108-z.

## Introduction

Current guidelines recommend the routine performance of percutaneous coronary angioplasty (PCI) of significantly stenosed non-culprit vessels, in patients with STEMI and multi-vessel disease.^1^ Non-culprit PCI may be performed either at the time of primary PCI for STEMI, or up to 45 days after the procedure, without the need for documentation of ischemia. These recommendations are based on evidence from about 7000 patients enrolled in randomized trials comparing routine non-culprit PCI to guideline directed medical therapy.^2^ The principal benefits of routine non-culprit PCI in meta-analyses of these trials were a reduction in the risk of re-infarction and repeat revascularization. There was a reduction in cardiovascular mortality, but there was no significant effect on all-cause mortality.^2^ Though these data are compelling, one important limitation of the existing data has not been widely acknowledged. It is unclear from the published reports if patients in the culprit-only PCI arms of these trials underwent routine evaluation for inducible ischemia following primary PCI. On the contrary, in some studies, the thresholds for revascularization were kept high, despite the presence of objective evidence of ischemia.^3^ Such an approach may deny many patients timely, appropriate revascularization, and may increase the risk of ischemic events. On the other hand, angiographically significant lesions in non-culprit vessels, may not be functionally significant, and consequently, many PCI procedures performed without assessment of ischemia may be unnecessary. This is supported by findings from previous trials using FFR to guide revascularization of non-culprit coronary lesions. Nearly half the patients in the Compare Acute trial,^4^ and 31% in the DANAMI-3-PRIMULTI trial,^5^ had an FFR > 0.80, and did not undergo non-culprit PCI. Likewise, in the FLOWER-MI trial, 44% of angiographically significant lesions had an FFR > 0.80.^6^ Therefore, a strategy to identify physiologically significant lesions in non-culprit vessels which might benefit from revascularization, may be more efficient than routine PCI of all anatomically significant non-culprit lesions. We hypothesized that a strategy of systematic non-invasive assessment of inducible ischemia to guide decisions regarding non-culprit PCI, will be non-inferior to routine non-culprit PCI, in reducing ischemia burden.

## Methods

The Value of Gated-SPECT MPI for Ischemia-Guided PCI of non-culprit vessels in STEMI Patients with Multi vessel Disease after primary PCI (IAEA SPECT STEMI) trial was an international, randomised, non-inferiority trial, comparing a strategy of ischemia guided non-culprit vessel PCI to a strategy of routine non-culprit vessel PCI, among patients with STEMI and multi-vessel disease. The study was initiated and funded by the International Atomic Energy Agency. The study rationale and design have been outlined previously.^7^ The trial is registered in the Clinical Trials Registry, India (CTRI/2018/08/015384) which is a primary register of the International Clinical Trials Registry Platform (ICTRP) (http://www.who.int/ictrp/search/en/). Patients were recruited from clinical centres in nine countries: Brazil, Cuba, India, Mexico, Pakistan, Serbia, Singapore, Spain, and Turkey. The Nuclear Medicine division at the University of Brescia, Italy was the central core lab for the trial. Investigators at the Indian Institute of Public Health, Delhi, India devised the randomisation scheme, developed the electronic case record forms, managed the data, and performed the statistical analysis.

### Participants

Patients over the age of 18, presenting with a first STEMI, had a successful primary PCI, and had a significant stenosis (≥ 70% diameter stenosis) in at least one non-infarct-related coronary artery, or major side branch (≥ 2.5 mm diameter), were eligible to participate. We excluded patients with ≥ 50% stenosis of the left main coronary artery, or had TIMI flow < 3 in a stenosed non-culprit vessel, or had complex lesion anatomy (as judged by the treating interventionist) that would preclude complete revascularisation. We also excluded patients who were hemodynamically unstable, had mechanical complications, or had prior revascularisation with PCI or CABG.

### Randomisation, data collection and data management

Sequentially numbered opaque sealed envelopes were provided to the nuclear medicine departments at the recruiting centres. Envelopes were prepared at the central data management centre at the Indian Institute of Public Health, Delhi based on the sequence generated using stratified block randomization using Stata 13 (College Station, TX). Clinical sites were the stratifying factor. When an eligible and consenting, patient was identified by the interventional cardiologist, the nuclear physician opened the envelope to reveal the management strategy (ischaemia guided or routine non-culprit PCI). Data were entered into a validated clinical data management system with inbuilt checks and audit trail (Clinion, https://www.clinion.com/electronic-data-capture-edc-software/).

### Study procedures

#### Primary angioplasty and non-culprit PCI

Patients underwent primary PCI with placement of stents as per local hospital practices. Patients randomized to the routine non-culprit PCI arm underwent PCI of the angiographically significant lesions (≥ 70% diameter stenosis) in non-culprit vessels either immediately (during the index primary PCI), or as a staged procedure preferably within 6 weeks after the index PCI. Patients in the ischemia guided arm underwent PCI to non-culprit vessels if the stress MPI showed at least mild inducible ischemia as determined by an SDS ≥ 4. Ischemia testing was not required if patients were symptomatic. Investigators were encouraged to perform the PCI as early as possible after randomisation. The choice of stents, antiplatelet agents and adjunctive therapies were left to the discretion of the treating cardiologist, in keeping with international guidelines and local practices.

#### Stress myocardial perfusion imaging

Patients randomised to the ischemia-guided arm underwent vasodilator stress testing within 7 days of the index STEMI. Gated-SPECT MPI was performed using a 1 or 2-day pharmacological (vasodilator) stress-rest or rest-stress protocol with 99mTc-sestamibi or 99mTc-tetrofosmin. Vasodilator stress agents used were dipyridamole, adenosine or regadenoson. At all participating sites cardiac stress procedures, acquisition and processing of MPI were performed as per ASNC guidelines.^8,9^ Patient management decisions were made based on the results of MPI as interpreted locally. Anonymized MPI files were submitted to the imaging core lab for processing and quantification of ischemia.

#### Image processing and analysis at the core lab

If the participating centers adopted resolution recovery acquisition protocols (GE Evolution for Cardiac, Philips Astonish, Siemens IQ SPECT or D-SPECT), they uploaded both raw data (for quality control) and reconstructed data for processing. Data submitted to the core lab were de-identified, and the core lab readers were unaware of treatment allocation. Quality control of the raw data was performed for all studies. Collected counts, presence of artefacts, patient motion, presence of interfering visceral activity, its intensity and distance from myocardial wall, detectable attenuation artifacts were all taken into consideration in determining ischemic burden. The core lab processed the DICOM data in a standard format for qualitative analysis. The SPECT images were reconstructed using an Iterative Reconstruction Algorithm, and the INVIA Corridor 4DM v2017 software was adopted for semi-quantitative analysis, providing perfusion defect scores (SSS, SRS, SDS), and perfusion defects extent globally and for specific vascular territories using a 17-segment model.^10^ The semi-quantitative analysis was performed using the normal databases that the software package implements for each acquisition system (General Electric, Philips, Siemens, DPSECT). Two expert readers (RG and CAL) carried out the final evaluation of the analysis score (SSS, SRS), taking into consideration all artefacts and processing errors.

#### Outcomes

The primary outcome was the difference in the percent ischemic myocardium at follow-up imaging between the two arms. The percentage of ischemic myocardium was calculated by subtracting the total perfusion defect (TPD) at rest from the TPD at stress. However, on review of the images the core lab detected artefacts in several cases and advised to use an alternative approach to calculate the amount of ischemic myocardium from the corrected SDS values. For this purpose, we used the approach proposed and validated by Berman et al^11^ Briefly, the total reversible perfusion defect (TPD) was quantified as SDS/68 × 100 (%), where SDS = SSS-SRS, as applied to a 17-segment model (68 = 17 × 4, and 4 is the maximal possible perfusion score for each segment). The parameters were derived automatically and corrected after visual inspection for imaging artefacts. Death, non-fatal myocardial infarction, and hospitalization were key secondary outcomes.

### Sample size considerations and statistical analysis

Based on the nuclear sub study of the Clinical Outcomes Using Revascularization and Aggressive Drug Evaluation (COURAGE) trial, we anticipated that the average percentage of ischemic myocardium at follow-up imaging would be about 5% in either arm (SD ± 7%). Based on this, we estimated that a total sample size of 100 patients would provide over 95% power for a non-inferiority margin (delta) of 5% at a one-sided alpha of 2.5% (if the SD of the difference in % ischemic myocardium was 7 or lower). The primary analysis was a modified intention-to-treat analysis, which excluded patients who did not have the follow-up MPI (either due to death or loss to follow-up). No imputation was performed to account for missing data. Per-protocol analyses excluded patients who crossed over, and those in the ischemia-guided arm, who did not undergo baseline MPI. Since the mean difference in percent ischemia was not normally distributed, quantile regression was performed to compare the median percentage ischemia between the two arms. Bootstrapping with 50 repetitions was used to generate 95% confidence intervals. We inferred non-inferiority of ischemia-guided PCI over routine non-culprit PCI, if the upper limit of the 95% CI of this difference was < 5%. In a sensitivity analysis, we also used a generalised linear model using the gamma distribution with an identity link, to compute mean differences and the 95% CI. All analyses were adjusted for clinical site as a stratifying variable. Analyses were performed using Stata 17 (College Station, TX).

## Results

Between August 2018 and February 2021, 109 patients were enrolled at nine participating hospitals. The baseline characteristics of enrolled patients are presented in Table 1. Over a third of patients were Hispanic, and over a fifth were of South Asian ethnicity. Patients were predominantly male, and had a high burden of atherosclerotic risk factors, with over a third having diabetes, and nearly 45% being smokers (Table 1). The majority of patients (105/109, 98%) underwent primary PCI within 12 h after symptom onset, with 80% receiving a drug-eluting stent. TIMI 3 flow was achieved in over 96% of patients. Baseline characteristics between the two randomised groups were similar except for a larger proportion of anterior STEMIs (LAD or diagonal as the infarct related artery) in the ischemia-guided PCI arm (Table 1).

**Table 1:** Baseline characteristics of included patients

	Ischemia-guided PCI N = 53	Routine NCPCI N = 56
Age (mean, standard deviation)	59.1 (10.8)	58.8 (11.2)
Males (n, %)	42 (79.3)	42 (75)
Ethnicity (n, %)		
South Asian	14 (26.4)	15 (26.8)
Caucasian	20 (37.7)	23 (41.1)
Hispanic	19 (35.9)	15 (26.8)
Black	0 (0)	3 (5.4)
Risk factors (n, %)		
Diabetes	20 (37.7)	18 (32.1)
Hypertension	33 (62.3)	32 (57.1)
Dyslipidemia/Statin treatment	23 (43.4)	15 (26.8)
Smoking	23 (43.4)	25 (44.6)
Family history of premature CAD	12 (22.6)	12 (21.4)
STEMI localisation (n, %)		
Anterior	26 (49.1)	15 (26.8)
Anterolateral	2 (3.8)	3 (5.4)
Inferior	22 (41.5)	35 (62.5)
High lateral	3 (5.7)	3 (5.4)
True posterior	0 (0)	0 (0)
Time from symptom onset to hospital arrival (hours) (median, IQR)	2.9 (3.5)	2.4 (2.6)
Door to balloon time (hours) (median, IQR)	1.5 (1.1)	1.3 (0.8)
Symptom to balloon time (hours) (median, IQR)	4.9 (4.2)	3.6 (4.4)
PPCI details (n, %)		
Transradial approach	32 (60.4)	34 (60.7)
Infarct related artery (n, %)		
LAD or LCX/OM/PDA	28 (52.8)	20 (35.7)
RCA/PDA/PLV	20 (37.7)	27 (48.2)
Ramus/Diagonal	5 (9.4)	9 (16.1)
Pre procedure IRA flow (n, %)		
TIMI 0	43 (81.1)	46 (82.1)
TIMI 1	4 (7.6)	3 (5.4)
TIMI 2	5 (9.4)	2 (3.6)
TIMI 3	1 (1.9)	5 (8.9)
Number of stents (n, %)		
0	1 (1.9)	1 (1.8)
1	37 (69.8)	42 (75)
2	14 (26.4)	9 (16.1)
3	1 (1.9)	4 (7.1)
Type of stent (n, %)		
Bare metal stent	13 (25)	9 (16.4)
Drug eluting stent	39 (75)	46 (83.6)
Post procedure IRA flow (n, %)		
TIMI 2	2 (3.8)	2 (3.6)
TIMI 3	51 (96.2)	54 (96.4)
Adjuvant therapy (n, %)		
Bivalirudin	1 (1.9)	1 (1.8)
Gp IIb–IIIa antagonists	11 (20.8)	12 (21.4)
Medications at discharge (n, %)		
Aspirin	51 (96.2)	55 (98.2)
Clopidogrel	29 (54.7)	32 (57.1)
Ticagrelol/Prasugrel	20 (37.7)	24 (42.9)
Statins	49 (92.5)	52 (92.9)
ACE inhibitor	42 (79.3)	39 (69.6)
Angiotension receptor blocker	2 (3.8)	4 (7.1)
Beta-blocker	41 (77.4)	50 (89.3)
Calcium channel blocker	5 (9.4)	7 (12.5)
Nitrates	14 (26.4)	6 (10.7)
Diuretics	15 (28.3)	16 (28.6)
Insulin	12 (22.6)	10 (17.9)
Oral hypoglycaemic agents	14 (26.4)	13 (23.2)

*PCI*, percutaneous coronary angioplasty; *NCPCI*, non-culprit percutaneous coronary angioplasty; *PPCI*, primary percutaneous coronary angioplasty; *CAD*, coronary artery disease; *LAD*, left anterior descending coronary artery; *LCX*, left circumflex coronary artery; *OM*, obtuse marginal coronary artery; *RCA*, right coronary artery; *PDA*, posterior descending coronary artery; *PLV*, posterior left ventricular artery; *TIMI*, thrombolysis in myocardial infarction flow grade

**Figure 1:**
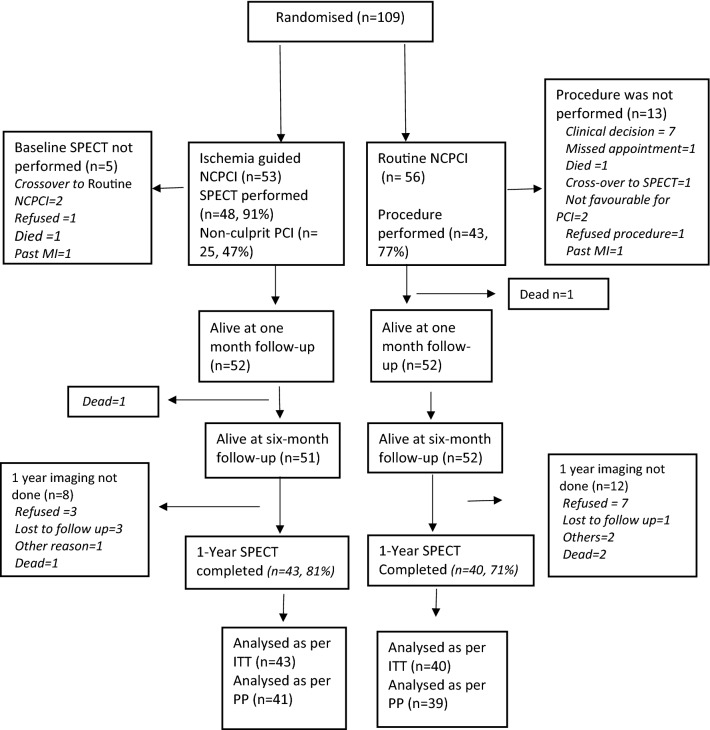
CONSORT trial flow chart

Both patient recruitment and initial and follow-up MPI were affected by the Covid-19 pandemic. Baseline stress MPI was performed in 48/53 (91%) of eligible patients at a median of 5 days after randomisation. The results of the baseline imaging studies are presented in Table S1 in the appendix. Of these, 25 patients (47%) underwent PCI to non-culprit vessels. Ninety percent of the non-culprit vessel PCIs were performed within 120 days of randomisation. Seven patients in the routine non-culprit PCI arm did not undergo the procedure on the advice of their treating cardiologist. In all, 43 patients (77%) underwent routine non-culprit PCI, with 37 (86%) undergoing the procedure within 6 weeks of randomisation (Figure 1). Details of non-culprit PCI are presented in Table S2 in the appendix. Follow-up SPECT MPI was performed at about 16.5 months. The planned schedule of 1-year imaging could not be adhered to in most countries because of the Covid-19 pandemic related restrictions. Core lab images were available for 43/53 (81%) patients in the ischemia-guided non-culprit PCI arm, and for 40/56 (71%) of patients in the routine non-culprit PCI arm (Figure 1). Adenosine was used in over half the studies, and dipyridamole and regadenoson in the remaining.

### Study outcomes

The results of the stress perfusion studies are summarized in Table S2 in the appendix. The median percent of ischemic myocardium in the baseline stress MPI was 4.4% among patients in the ischemia-guided arm, with about 13% (6/48) patients having ischemia involving ≥ 10% of the myocardium. On follow-up imaging, this reduced to 2.9%, with 7% (3/43) having ≥ 10% ischemic myocardium. The median percentage of ischemic myocardium on follow-up imaging was identical (2.9%) in both arms. The median difference (adjusted for site) was clinically insignificant, and the upper limit of the 95% confidence interval was 1.6, which was less than the pre-specified non-inferiority margin of 5% (Table 2). Analysis using the mean values for percent ischemic myocardium and their difference also showed similar results. A total of 7 patients died, 3 in the ischemia-guided and 4 in the routine non-culprit PCI arm. One death was attributable to Covid-19, and the remaining were categorized as being due to cardiovascular causes by investigators. In addition, 2 patients in the ischemia-guided arm had a non-fatal MI and unstable angina. One patient in the routine non-culprit PCI arm suffered a non-fatal MI. No repeat re-intervention procedures were performed in either arm.

**Table 2:** Study outcomes

Outcome	Ischemia-guided PCI arm (n = 43)	Routine non-culprit (n = 40)	Adjusted* median difference (quantile regression) (95% CI)	Adjusted* median difference (bootstrapped** estimates) (95% CI)	*P* value for non-inferiority
Median % ischemic myocardium (interquartile range)-intention-to-treat analysis^¶^	2.94 (0, 5.9)	2.94 (0, 4.41)	0.13 (− 1.32, 1.60)	0.13 (− 1.30, 1.57)	< .0001
Median % ischemic myocardium (interquartile range)-per-protocol analysis	2.94 (0, 4.41)	2.94 (0, 4.41)	0.08 (− 1.38, 1.54)	0.08 (− 1.10, 1.26)	< .0001
Clinical outcomes					
Death	3	4	–	–	–
Non-fatal MI	2	1	–	–	–
Unstable angina	2	0	–	–	–

*Adjusted for site as fixed effect, **Bootstrapping with 50 repetitions

^¶^Results using a generalised linear model with gamma distribution yielded similar results: (Mean difference 0.19, 95% CI − 1.67 to 2.05)

## Discussion

In this international, randomized trial, we found that patients who underwent ischemia-guided PCI of non-culprit vessels had low ischemia burden on follow-up imaging, which was similar to that in those who underwent routine non-culprit PCI. The absolute difference in the percentage of ischemic myocardium was small, and the ischemia-guided strategy was non-inferior to routine non-culprit PCI at the pre-specified margin. These results are based on standardized image processing and interpretation at a central core lab, which was blinded to treatment allocation.

The management of significantly stenosed non-culprit vessels in patients presenting with STEMI and multi-vessel disease, has evolved rapidly. Meta-analyses of recent randomized trials suggest that there is a reduction in the risk of re-infarction and repeat revascularization among patients undergoing routine PCI for non-culprit vessels.^2,12^ Although there was no reduction in all-cause mortality, there was a reduction in cardiovascular deaths. A major limitation of these trials however, is that, patients in the culprit-only PCI arms of these studies did not undergo systematic evaluation for ischemia, and some studies had a high threshold for revascularization even for patients with objective evidence of ischemia.^3^ For example, in the largest such trial, Complete vs Culprit-Only Revascularization Strategies to Treat Multivessel Disease after Early PCI for STEMI (COMPLETE), patients in the culprit-only PCI arm were prescribed medical therapy “with no further revascularization, regardless of whether there was evidence of ischemia on non-invasive testing”.^3^ Patients underwent PCI of non-culprit vessels on follow-up only if they had “intractable angina (CCS class 3 or 4)” despite guideline directed medical therapy, *and* also had documented evidence of ischemia in the territory of the non-culprit vessels.^3^ This could have partly contributed to the increase in the risk of repeat revascularization and re-infarction, seen among patients who were randomized to culprit-only PCI.

**Figure 2:**
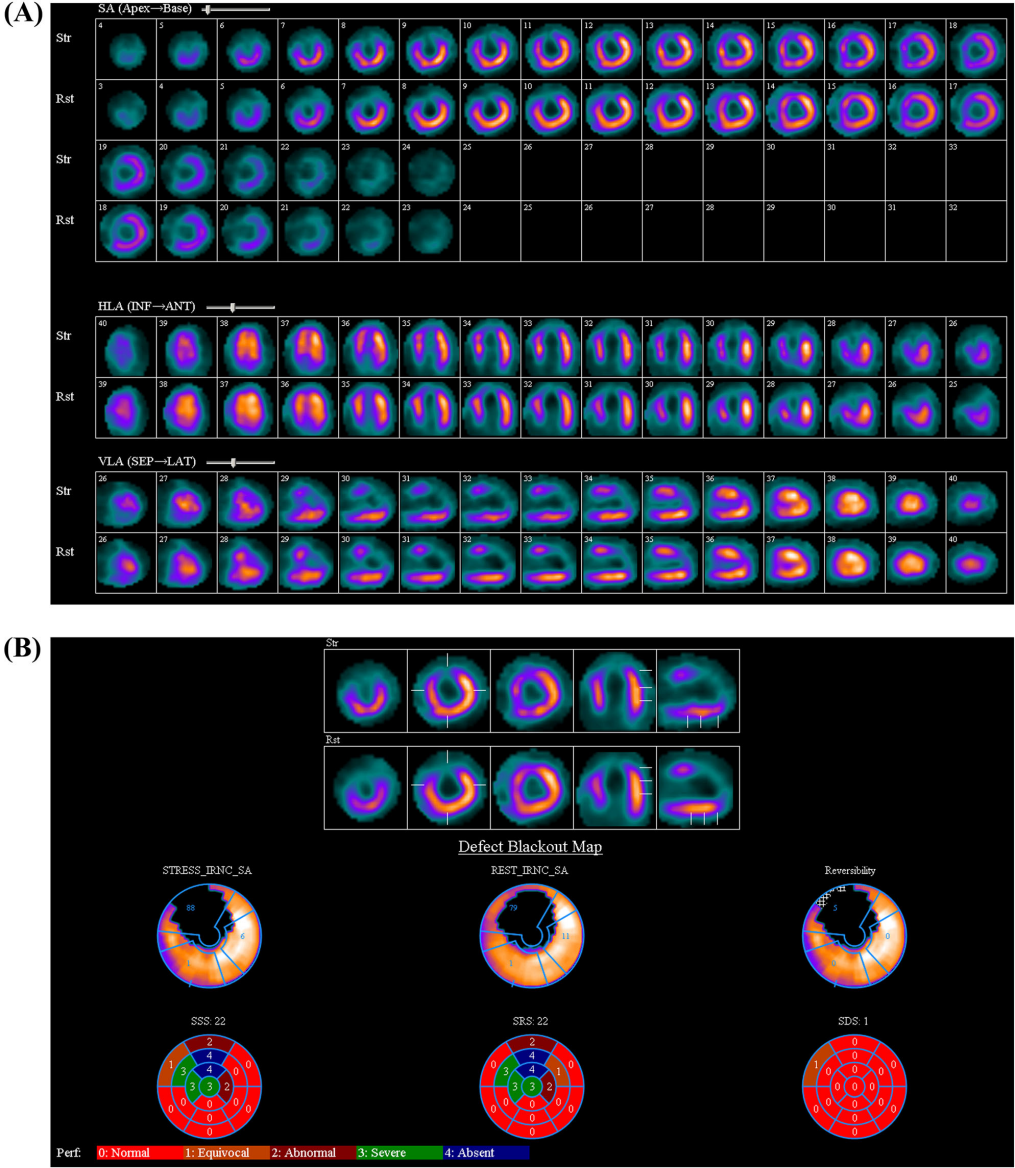

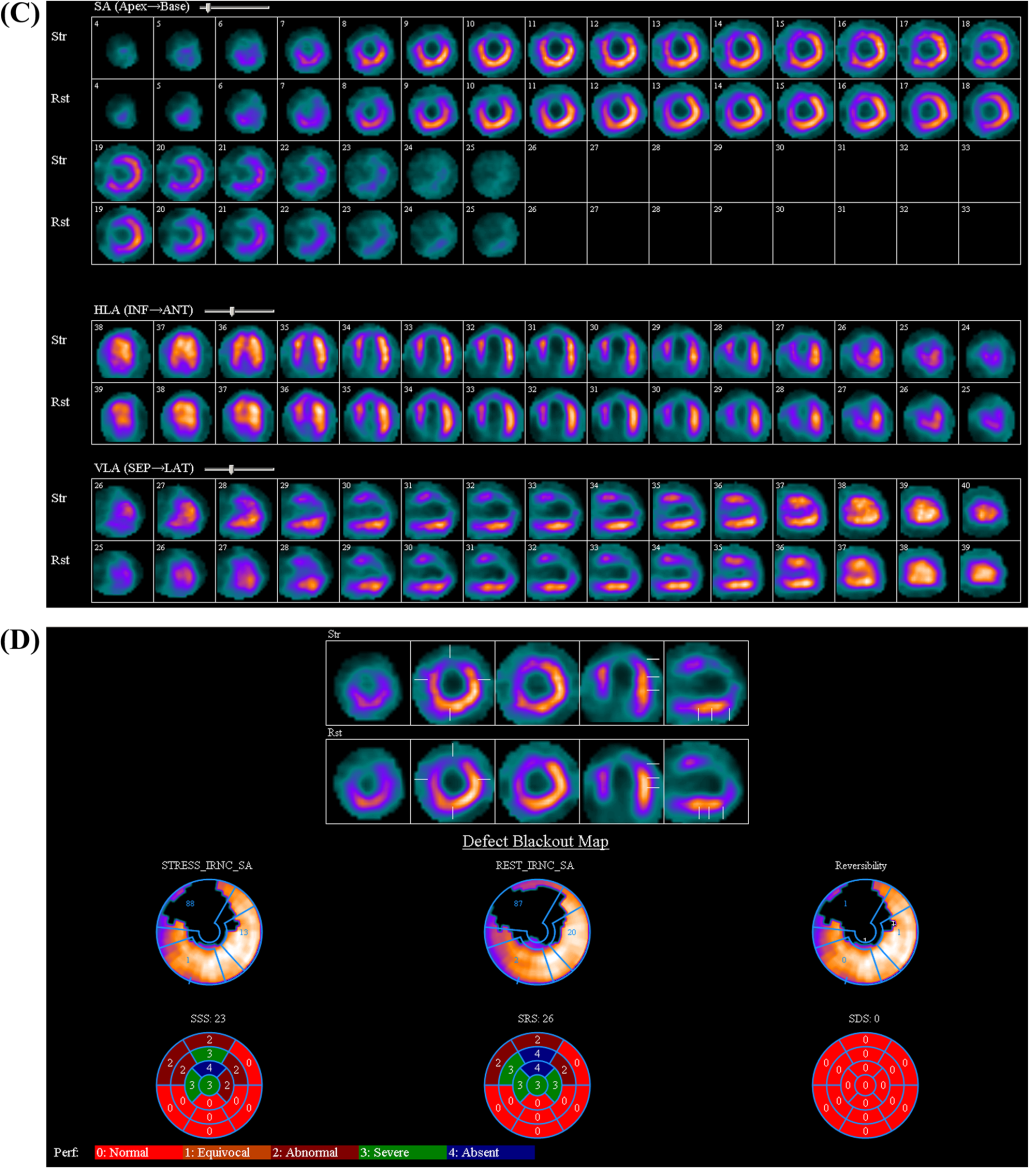
Not all angiographically stenosed non-culprit vessels need treatment. Baseline and follow-up stress MPI of a 44 year-old male smoker who presented with anterior wall STEMI and underwent primary PCI to the LAD. He had 90% stenosis of both the LCx and the RCA. (**A**) Stress/Rest MPI in short axis (SA), Horizontal long axis (HLA) and vertical long axis. There is an irreversible perfusion defect involving anterior and apical walls. (**B**) The SDS was considered equal to 0, TID was 0.97. Patient did not undergo non-culprit PCI. (**C**, **D**) Results at follow-up imaging were unchanged. *STEMI-ST* segment elevation myocardial infarction, *PCI* percutaneous coronary intervention, *LAD* left anterior descending coronary artery, *LCx* left circumflex coronary artery, *RCA* right coronary artery, *MPI* myocardial perfusion imaging, *SDS* summed difference score, *TID* transient ischemic dilatation

In addition, a strategy of treating all non-culprit vessels without regard to the presence of ischemia may result in a number of patients undergoing unnecessary revascularization procedures (Figure 2). Evidence for this assertion comes from the trials which used FFR to determine the necessity of non-culprit vessel PCI following STEMI.^4–6^ In these studies, about 30%–50% of patients had FFR values > 0.80 in the non-culprit vessels, thereby obviating the need for PCI. Nevertheless, using ischemia guidance preserved the benefits over culprit-only PCI, in terms of a reduction in risk of the composite of death, MI or repeat revascularization.^4,5,12^ Likewise, in this study, only 25 of 48 (47%) patients required non-culprit PCI based on the results of ischemia testing. Despite this, there were no meaningful differences in ischemia burden on follow-up imaging at 16.5 months. These results are consistent with those of the Flow Evaluation to Guide Revascularization in Multivessel ST-Elevation Myocardial Infarction (FLOWER-MI) trial.^6^ In this study, a strategy of routine non-culprit PCI, failed to show a difference in the composite of all-cause death, non-fatal MI or unplanned hospitalization leading to revascularization, when compared to an ischemia-guided (using FFR) approach.^6^

We used vasodilator stress MPI for ischemia guidance in this trial based on several considerations. First, data from previous studies have shown that it is feasible, and safe to perform vasodilator stress testing within 10 days after STEMI.^13^ Second, vasodilator MPI provides useful prognostic information that helps risk-stratify patients,^14^ and may aid in decisions regarding non-culprit PCI. And, finally, FFR is expensive, and may not be readily available, particularly in LMICs. We reasoned that a strategy that employs a less costly non-invasive test, may be more applicable to low-resource settings.

### Strengths and limitations

The main strengths of our study are that it is a randomized controlled trial, involving participants from nine countries, encompassing a wide range of country-income levels and healthcare systems. We used reports generated by a blinded core lab for all analyses. An important limitation of this study is that just over three-fourth of patients in the routine non-culprit PCI arm underwent revascularization. However, this decision was primarily taken by the treating cardiologist based on clinical need, and the ischemia burden remained low at study end. We enrolled a small number of patients, and our study is not powered to detect differences in clinical outcomes. Nevertheless, the sample size was sufficient to demonstrate non-inferiority on the primary outcome with sufficient precision and confidence. Finally, the reduction in cardiac imaging and procedure volumes seen during the Covid-19 pandemic,^15^ affected the timeliness of follow-up, performance of imaging, and PCI in this study. However, the majority of patients completed the study procedures and were available for follow-up as restrictions eased.

## Conclusion

A consensus has now emerged regarding the need to revascularize non-culprit vessels following primary PCI.^1^ We believe that the next critical question is whether this approach can be further refined by evaluating if a strategy of ischemia-guided non-culprit vessel PCI, performs as well as routine non-culprit PCI. A selective approach to treating non-culprit lesions following primary PCI, has the potential to reduce costs and complications, without adversely affecting outcomes. Such a strategy may be particularly relevant to resource-limited settings in low and middle income countries (LMIC). The results from the IAEA SPECT STEMI trial provide encouraging preliminary data in this regard.

## New knowledge gained

These preliminary data suggest that there is potential value in a strategy of systematic, ischemia-guided management of patients with multivessel disease after primary PCI for STEMI.

## Supplementary Information

Below is the link to the electronic supplementary material.

Supplementary file1 (DOCX 17 KB)

Supplementary file2 (PPTX 345 KB)

## Abbreviations

STEMI-ST: Segment elevation myocardial infarction
PCI: Percutaneous coronary intervention
LAD: Left anterior descending coronary artery
LCx: Left circumflex coronary artery
RCA: Right coronary artery
PDA: Posterior descending coronary artery
MPI: Myocardial perfusion imaging
SDS: Summed difference score
TID: Transient ischemic dilatation
TIMI: Thrombolysis in myocardial infarction flow grade
