# Supplementary material for: Ischemia-guided vs routine non-culprit vessel angioplasty for patients with ST segment elevation myocardial infarction and multi-vessel disease: the IAEA SPECT STEMI trial
Source: J Nucl Cardiol. 2022 Oct 25;30(3):1091–102. doi: 10.1007/s12350-022-03108-z (PMC9595582; doi:10.1007/s12350-022-03108-z)
Supplement: Supplementary file 1 — Supplementary file1 (DOCX 17 KB) [file 12350_2022_3108_MOESM1_ESM.docx]

**Supplementary appendix**

**Ischemia-guided versus routine non-culprit vessel angioplasty for patients with ST segment elevation myocardial infarction and multi-vessel disease - the IAEA SPECT STEMI trial**

Karthikeyan et al.

**Table S1: Results of baseline imaging**

| Variable, | **Clinical site recording** | **Core lab reading** | **Corrected for artefacts (Core lab)** |
| --- | --- | --- | --- |
| SSS | 13.7 (7.2) | 14.9 (9.8) | 14.8 (10.2) |
| SRS | 10.1 (6.8) | 12.5 (9.6) | 11.8 (9.5) |
| SDS Median (IQR) | 3.5 (3.2)  3 (1, 6) | 3.2 (3.6)  2 (1,5) | 2.9 (3)  3 (0,4) |
| Rest LVEF | 49.3 (13.2) | 55.6 (14.3) |  |
| Rest LVEDV | 97 (32.2) | 102.2 (37.8) |  |
| Rest LVESV | - | 48.3 (30.2) |  |
| Rest TPD | 17 (11.3) | 25.7 (24.2) |  |
| Stress LVEF | 48.3 (15.5) | 54.8 (14.5) |  |
| Stress LVEDV | - | 106.9 (37.9) |  |
| Stress LVESV | - | 51.4 (29.8) |  |
| Stress TPD | 18.3 (10.6) | 27.6 (19.6) |  |
| TID | - | 1.1 (0.16) |  |

**Table S2: Results of follow-up imaging**

|  | **Clinical site,** Mean (SD) | | **Core lab,** Mean (SD) | |
| --- | --- | --- | --- | --- |
|  | **Ischemia-guided PCI arm**  **n=43** | **Routine non-culprit PCI arm**  **n=40** | **Ischemia-guided PCI arm**  **n=43** | **Routine non-culprit PCI arm**  **n=40** |
| SSS | 9.3 (7.1) | 9.8 (8.5) | 11.3 (8.8) | 10.2 (9.3) |
| SRS | 6.5 (5.8) | 8.2 (8.5) | 9 (8.6) | 8 (8.8) |
| SDS | 2.7 (3.7) | 1.65 (2.7) | 2.3 (2.9) | 2.2 (3) |
| Rest LVEF | 53 (14.5) | 53 (13) | 57.1 (12.1) | 58 (11.6) |
| Rest LVEDV | 98.7 (39) | 100 (42.3) | 110.6 (42.4) | 105.6 (45.9) |
| Rest LVESV |  |  | 50.8 (31.8) | 46.6 (28) |
| Rest TPD | 9.4 (8.9) | 12 (11.4) | 17.5 (17.1) | 17.4 (16.8) |
| Stress LVEF |  |  | 56.4 (14.3) | 57.6 (12.3) |
| Stress LVEDV |  |  | 118 (49) | 112.6 (44.2) |
| Stress LVESV |  |  | 56.4 (41.7) | 50.8 (32) |
| Stress TPD | 11 (10.3) | 13.2 (12) | 22 (17.9) | 20.4 (17) |
| TID |  |  | 1.04 (0.15) | 1.04 (0.10) |

SSS-summed stress score, SRS-Summed rest score, SDS-Summed difference score, LVEF-Left ventricular ejection fraction, LVEDV-Left ventricular end-diastolic volume, LVESV-Left ventricular end-systolic volume, TPD-Total perfusion defect, TID-Transient ischemic dilatation

**Table S3: Details of non-culprit PCI in the two arms**

|  | **Ischemia guided Non culprit PCI** | **Routine NCPCI** |
| --- | --- | --- |
| Procedure done (n, %) | 25 (47.2) | 43 (76.8) |
| Number of days since randomisation (median, IQR) | 7 (22) | 7 (28) |
| **Arterial access (n, %)**  Transradial  Transfemoral | 19 (76)  6 (24) | 33 (76.7)  10 (23.3) |
| **Non-culprit vessel (n, %)**  LAD/Diagonal  LCX/OM/Left PDA/Ramus  RCA/PDA/PLV | 8 (32)  6 (24)  10 (40) | 21 (48.8)  9 (20.9)  14 (32.6) |
| **Pre procedure TIMI flow (n, %)**  0  1  2  3 | 1 (10)  2 (20)  2 (20)  5 (50) | 5 (35.7)  0 (0)  2 (14.3)  7 (50) |
| **Stents used (n, %)**  1  2  3 | 0 (0)  8 (80)  2 (20) | 2 (14.3)  12 (85.7)  0 (0) |
| **Type of Stent (n, %)**  Bare metal  Drug eluting | 9 (90)  1 (10) | 10 (83.3)  2 (16.7) |
