# Supplementary material for: Ischemia-guided vs routine non-culprit vessel angioplasty for patients with ST segment elevation myocardial infarction and multi-vessel disease: the IAEA SPECT STEMI trial
Source: J Nucl Cardiol. 2022 Oct 25;30(3):1091–102. doi: 10.1007/s12350-022-03108-z (PMC9595582; doi:10.1007/s12350-022-03108-z)
Supplement: Supplementary file 2 — Supplementary file2 (PPTX 345 KB) [file 12350_2022_3108_MOESM2_ESM.pptx]

## Slide 1
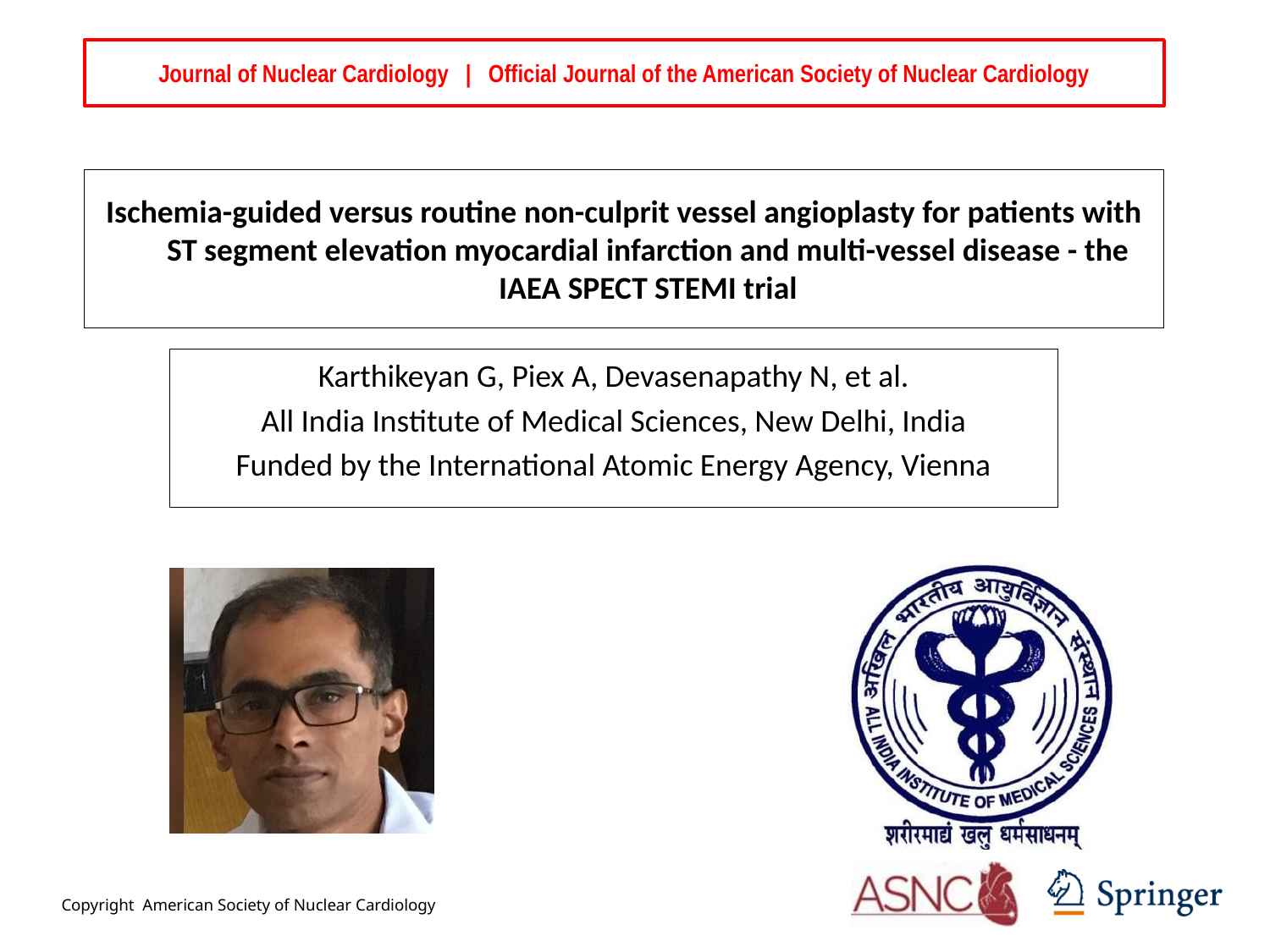

Journal of Nuclear Cardiology | Official Journal of the American Society of Nuclear Cardiology
# Ischemia-guided versus routine non-culprit vessel angioplasty for patients with ST segment elevation myocardial infarction and multi-vessel disease - the IAEA SPECT STEMI trial
Karthikeyan G, Piex A, Devasenapathy N, et al.
All India Institute of Medical Sciences, New Delhi, India
Funded by the International Atomic Energy Agency, Vienna
Copyright American Society of Nuclear Cardiology

## Slide 2
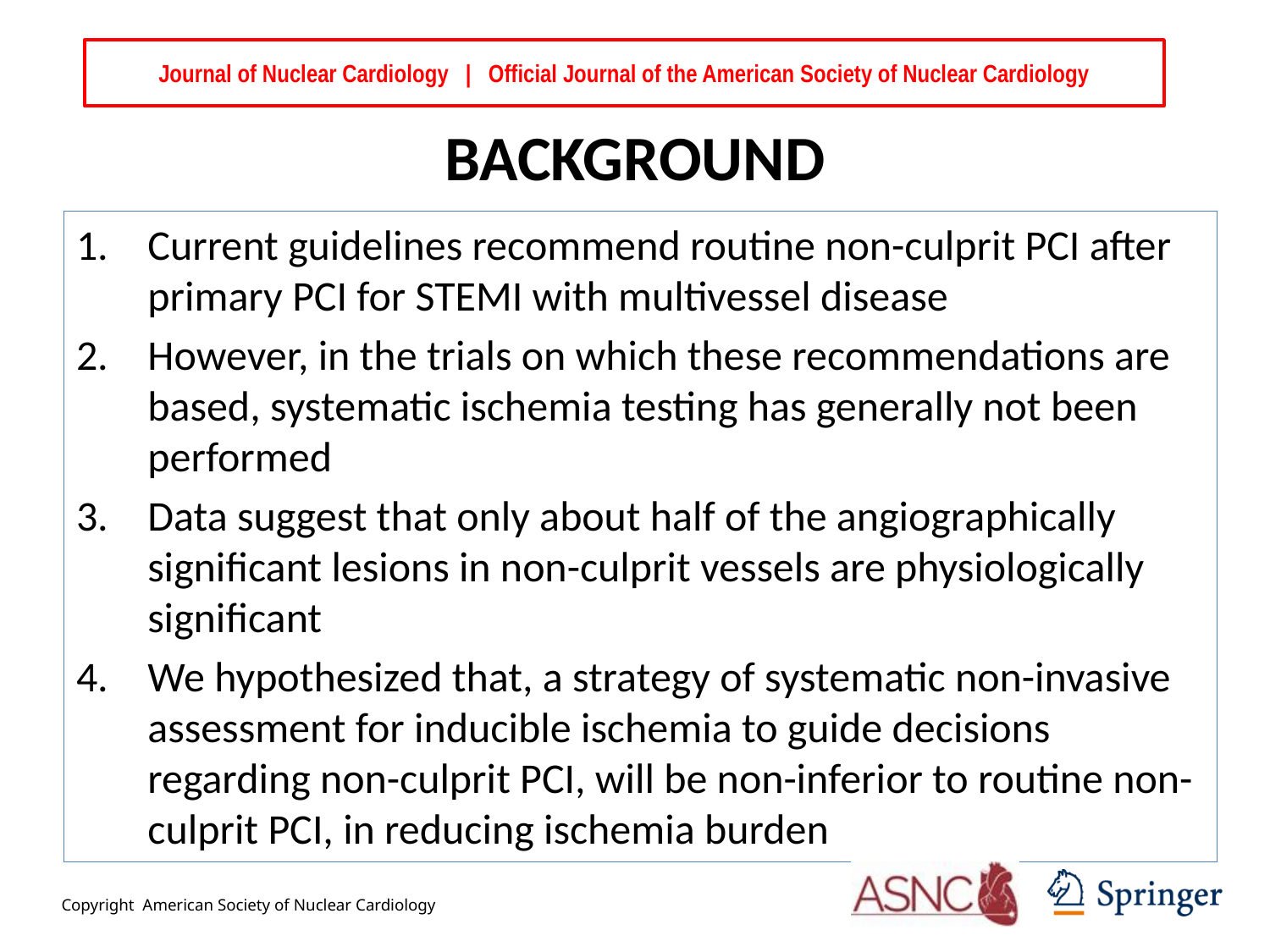

Journal of Nuclear Cardiology | Official Journal of the American Society of Nuclear Cardiology
# BACKGROUND
Current guidelines recommend routine non-culprit PCI after primary PCI for STEMI with multivessel disease
However, in the trials on which these recommendations are based, systematic ischemia testing has generally not been performed
Data suggest that only about half of the angiographically significant lesions in non-culprit vessels are physiologically significant
We hypothesized that, a strategy of systematic non-invasive assessment for inducible ischemia to guide decisions regarding non-culprit PCI, will be non-inferior to routine non-culprit PCI, in reducing ischemia burden
Copyright American Society of Nuclear Cardiology

## Slide 3
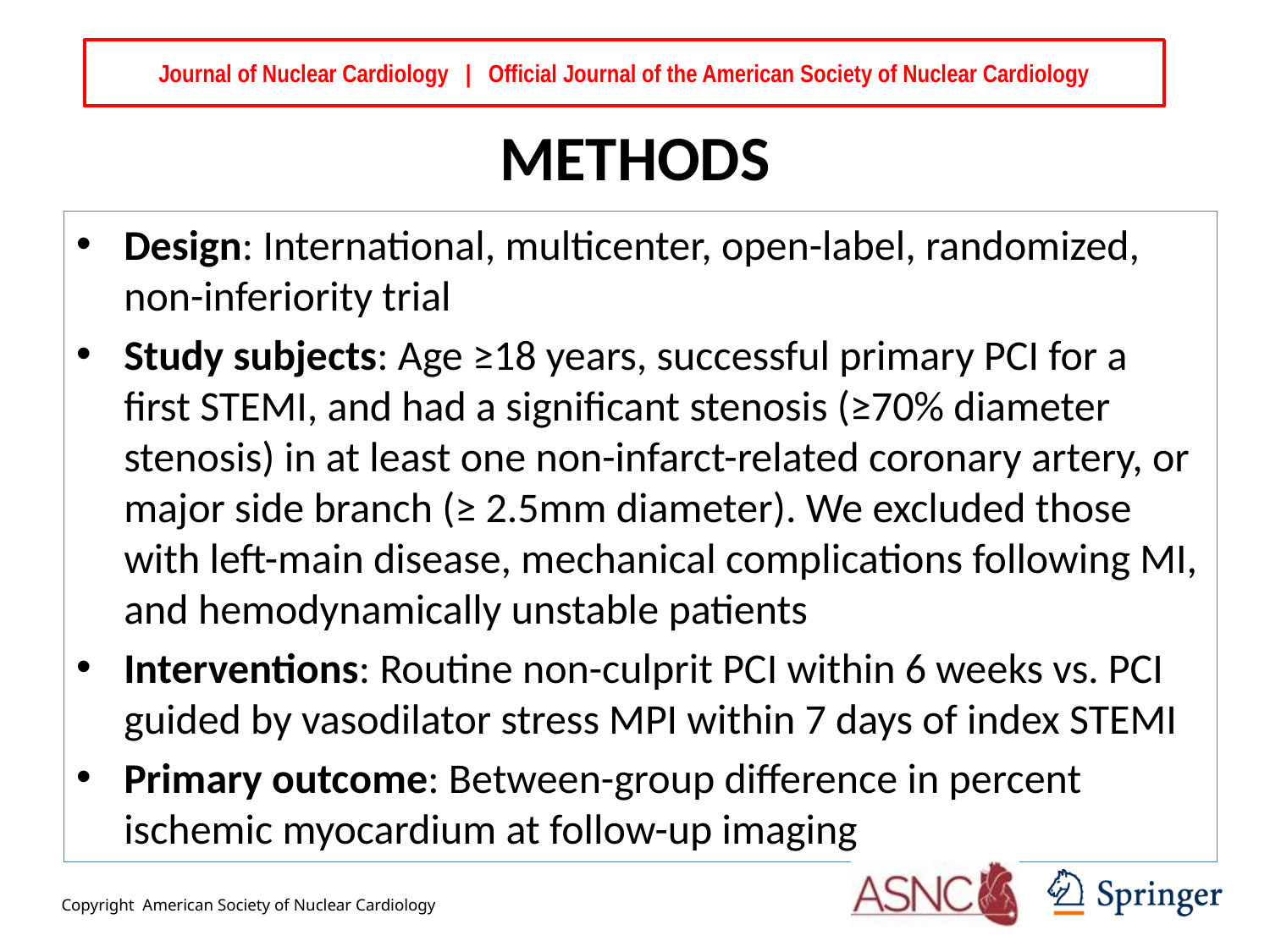

Journal of Nuclear Cardiology | Official Journal of the American Society of Nuclear Cardiology
# METHODS
Design: International, multicenter, open-label, randomized, non-inferiority trial
Study subjects: Age ≥18 years, successful primary PCI for a first STEMI, and had a significant stenosis (≥70% diameter stenosis) in at least one non-infarct-related coronary artery, or major side branch (≥ 2.5mm diameter). We excluded those with left-main disease, mechanical complications following MI, and hemodynamically unstable patients
Interventions: Routine non-culprit PCI within 6 weeks vs. PCI guided by vasodilator stress MPI within 7 days of index STEMI
Primary outcome: Between-group difference in percent ischemic myocardium at follow-up imaging
Copyright American Society of Nuclear Cardiology

## Slide 4
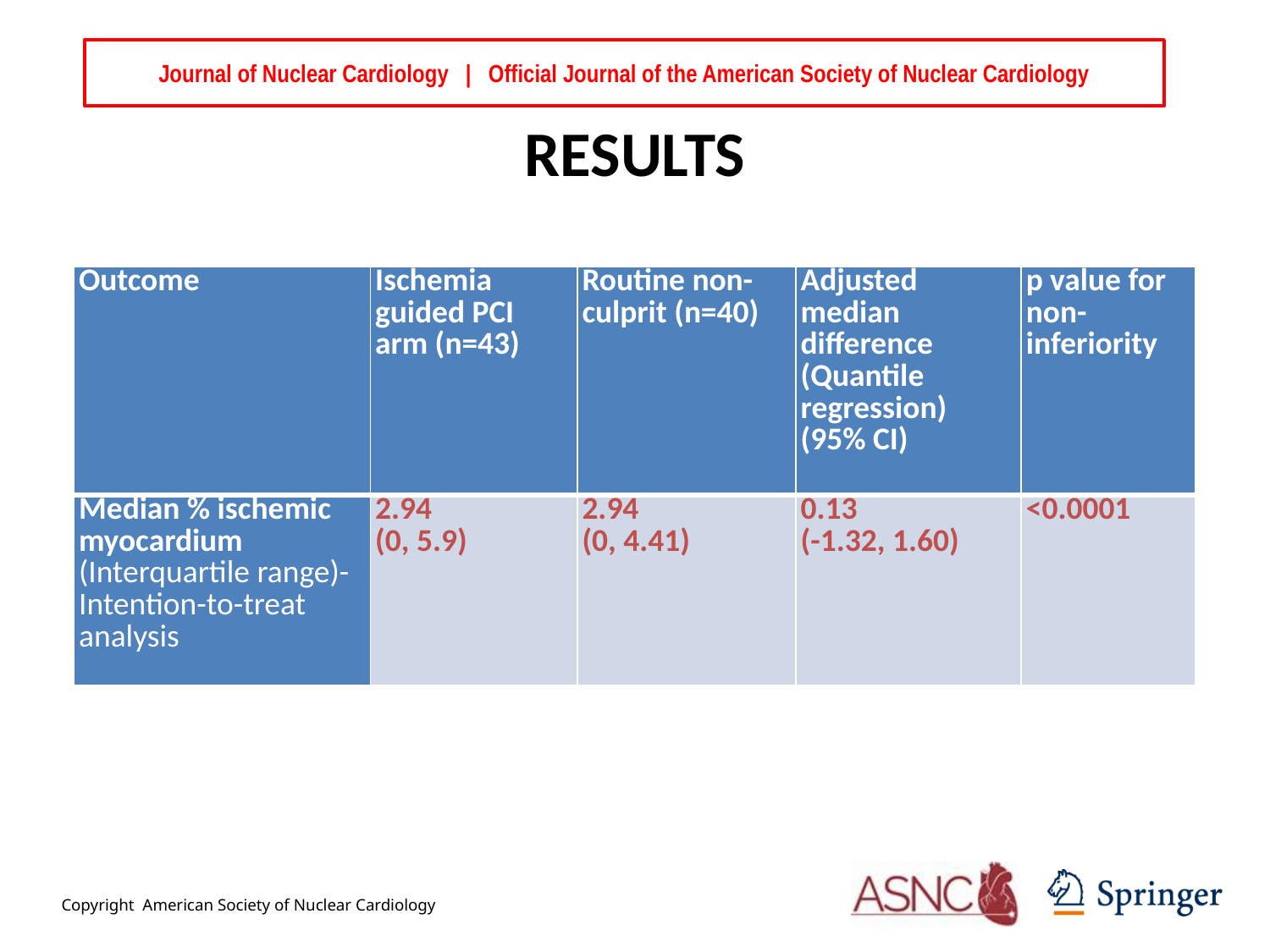

Journal of Nuclear Cardiology | Official Journal of the American Society of Nuclear Cardiology
# RESULTS
| Outcome | Ischemia guided PCI arm (n=43) | Routine non-culprit (n=40) | Adjusted median difference (Quantile regression) (95% CI) | p value for non-inferiority |
| --- | --- | --- | --- | --- |
| Median % ischemic myocardium (Interquartile range)- Intention-to-treat analysis | 2.94 (0, 5.9) | 2.94 (0, 4.41) | 0.13 (-1.32, 1.60) | <0.0001 |
Copyright American Society of Nuclear Cardiology

## Slide 5
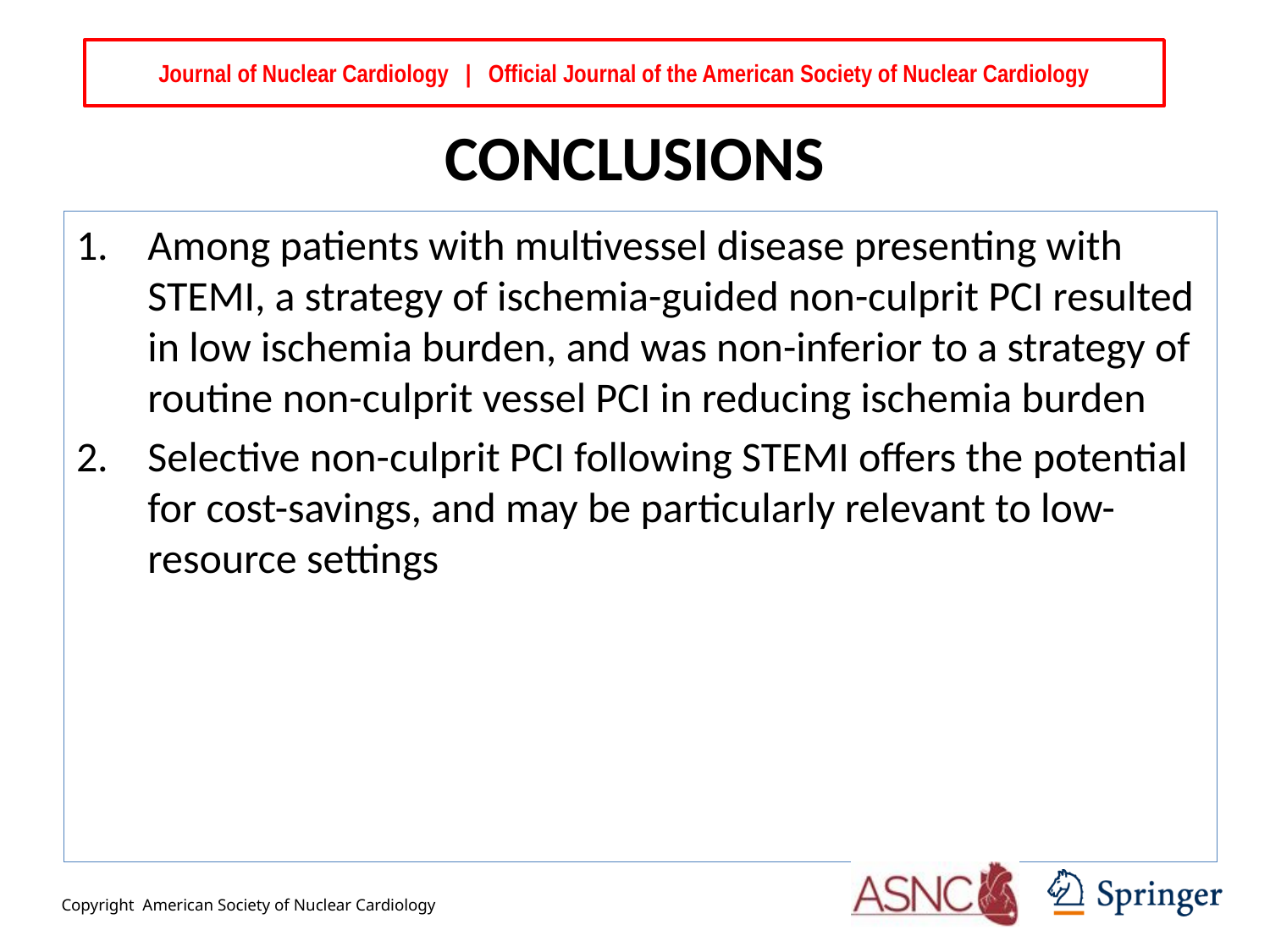

Journal of Nuclear Cardiology | Official Journal of the American Society of Nuclear Cardiology
# CONCLUSIONS
Among patients with multivessel disease presenting with STEMI, a strategy of ischemia-guided non-culprit PCI resulted in low ischemia burden, and was non-inferior to a strategy of routine non-culprit vessel PCI in reducing ischemia burden
Selective non-culprit PCI following STEMI offers the potential for cost-savings, and may be particularly relevant to low-resource settings
Copyright American Society of Nuclear Cardiology
